# What is risk? The challenge of defining ‘risk’ in caries risk assessment

**DOI:** 10.1080/00016357.2023.2275032

**Published:** 2024-03-26

**Authors:** Anna Senneby, Helena Fransson, the Foresight Research Consortium, Niklas Vareman

**Affiliations:** Department of Oral Radiology, Skåne University Hospital, Malmö, Sweden; Department of Endodontics, Faculty of Odontology, Malmö University, Malmö, Sweden; Listing of partners on https://mau.se/en/research/research-programmes/foresight/; Department of Medical Ethics, Lund University, Lund, Sweden

Dear Editor,

The primary goal of caries risk assessment is to identify individuals with increased risk of caries. Estimating an individual’s risk could help to target relevant courses of action for the patient—weighing the patient’s economic means, the discomfort of interventions, and the periodicity of treatment against the consequences of non-interventions. Central to the decision making, both for the clinician and the patient, is the extent of the consequences to the patients’ health. Accepting, as the authors of this text do, the view that decisions regarding interventions should be made jointly by the clinician and the patient, the clinician needs to communicate relevant information about the risk to the patient.

In order to do so, risk assessment of dental caries has to be a meaningful and effective process. First, there needs to be a clear understanding regarding the *definition* of risk. (The meaning of risk has to be clear to ensure that the result of a risk assessment is unequivocal to the clinician.) Second, risk *communication* between the clinician and the patient needs to be effective. Unless risk is defined, clinicians have to rely on their own intuitive understanding when communicating caries risk to patients, which may affect how patients respond to the results of the risk assessment. However, in many studies of risk assessment methods and models, risk is not defined. In a systematic review of different caries risk assessment methods, only one study included a definition of risk, whilst remaining studies were unclear or did not present any risk definitions [1].

## What is ‘risk’?

Risk researchers Terje Aven and Ortwinn Renn define, or characterize, risk as ‘uncertainty about and severity of the events and consequences (or outcomes) of an activity with respect to something that humans value’ [2]. That is, risk is a combination of what we *believe* about something happening and how we *value* that thing happening. Aven and Renn’s definition aims to formulate a basic intuition regarding risk, making it broad and in need of context.

Different fields focus on different aspects of the general conception of risk. Some focus on the *severity of outcomes* while others focus on the *ratios of unfavorable to favorable outcomes* or the *probability of outcomes of different levels of severity*. For example, in economics, portfolio theory defines risk as the variance of the portfolio [3]. In toxicological risk research, risk depends on the rate ratios of an event, for instance, in Vollset et al.’s study on cancer risk [4]. Risk can also be the value of an event happening weighted by the probability of it happening, as measured in any study of economic behavior, for instance, Kahnemann and Tversky’s study on decision making under risk [5], or simply the probability of some event happening, as in Reis and Román’s study on hazard and risk in environmental neurology [6].

Common to all is a need to identify the possibility of something bad happening in order to not only prepare in proportion to the risk but also find ways to make the risk smaller, if possible. Thus, risk assessment mainly concerns navigating towards an uncertain future.

With this in mind, we look at risk in a dentistry context and ask, how is caries risk defined? To answer this question, we examine two caries risk assessment tools: CAMBRA [7] and Cariogram [8].

## Caries risk definitions in two caries risk assessment tools

The two risk assessment tools CAMBRA and Cariogram have both been used to assess caries risk for over 20 years, and there are well over 100 research publications on each of them. In addition, they are both freely available to clinicians and represent tools from two continents: Europe (Cariogram) and North America (CAMBRA). Therefore, we believe they can provide different aspects and challenges regarding the definition of risk.

CAMBRA was developed based on research on key factors that contribute to the progression or prevention of caries. It provides caries risk assessment forms and instructions for the management of caries. It is a comprehensive program for assessing risk and, based on this assessment, determining interventions. By ticking off risk factors and protective factors and weighing them against each other, the tool provides a score for the severity of the risk [7]. The concept of ‘caries risk’ is actually not defined within CAMBRA; however, patients are sorted into risk groups that are defined as ‘Low’, ‘Moderate’, and ‘High’, and in one study [9], a fourth group was added: ‘Extreme’. Studies using CAMBRA discuss the outcome of one or more cavitated lesions [10] or approximal carious lesions radiographically [11] extending into the dentin after two years but the tool itself does not state any of these as an intended outcome.

Cariogram was developed as both a risk model and a prediction model, identifying risk factors and predicting those at high risk of disease [8]. It functions on a broad spectrum of populations with different caries prevalence rates and disease spectra. The tool offers an automated and weighted interpretation of available patient information, resulting in a percentage of chance of avoiding caries; the lower this percentage, the higher the risk of caries. In their original study, the developers of the tool do not address the concept of caries risk but deal directly with caries risk assessment:

The concept of caries risk assessment is, from one point of view, simple and straightforward. The idea is to: (a) identify those persons who will most likely develop caries and (b) provide these individuals proper preventive and treatment measures to stop the disease. [8]

However, their definition provides a distinction between those of high risk and those of no risk, which gives us an idea of what caries risk means. Studies on the accuracy of implementing Cariogram have defined outcomes over two and five years involving cavitated carious lesions or lesions within the dentine that would need restorative treatment [8]. The model only gives a probability for not acquiring caries at all and leaves it to the clinician to assess what the possible progression of the disease will look like in the individual case [12].

## Defining caries risk

**Figure 1:**
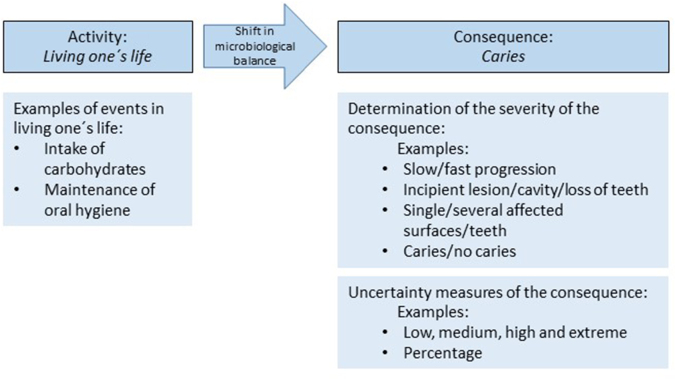
Simplified illustration of the main features of caries risk based on the definition proposed by Aven and Renn [2].

Aven and Renn’s general characterization of risk in terms of an activity’s outcomes and value indicates what we need to identify to define caries risk [2]. We need to define the activity, the outcomes of this activity, and the measures by which the uncertainty and severity of the outcomes are assessed (Figure 1).

### Activity and outcomes

Overall, there seems to be some confusion regarding the term ‘caries’ as it is used to refer both to the disease/process and to the outcome/consequence. Arguably, the latter should instead be referred to as, for example, carious lesion [13]. In this article, we have used the term as proposed in the cited literature—namely to refer to both process and outcome.

Regarding caries risk, the relevant activity can perhaps best be understood in a very broad sense as living one’s life. This can be narrowed down to a set of activities that are known, or suspected, to be conducive to caries, such as fermentable carbohydrate consumption and poor dental care. Choosing whether and how much one engages in these activities affects the outcome of interest: caries. Caries-relevant activities may be associated with other negative consequence; for instance, a high carbohydrate intake can lead to a number of negative consequences, such as diabetes or obesity. However, in this context, caries is the only one of interest.

Undoubtedly, caries as an outcome has value for patients, but is it tooth loss because of caries that is of interest when defining caries risk, or is it a cavity, pain, or simply finding the first miniscule sign of a possible enamel lesion? Reducing the outcomes to ‘caries’ is lumping together a wide array of distinct outcomes that may be valued differently by patients. Caries becomes ‘white spot’ OR ‘cavity’ OR ‘fractured tooth’ and so on; either one of these disjuncts is chosen to define the outcome caries if ‘caries’ is defined as the oral disease that is first detectable as white spot lesion on a tooth surface or something of that kind, or each disjunct can be an outcome of its own, leading to a risk assessment that looks at the possibility of each of these outcomes. Therefore, with the patients’ involvement in the decision making in mind, clinicians ought to think through and discuss the outcomes that would be most relevant if the patients are to make an informed decision regarding what interventions they can spend their resources on.

Neither CAMBRA nor Cariogram are very clear on the actual outcome. Although the architects of CAMBRA discuss the direction of the dysbiosis—that it ‘can be tipped toward caries progression and demineralization of the tooth mineral or toward repair of the tooth mineral by remineralization as a result of one or more protective factors’—the actual outcome and what they seem to want to avoid is not explicitly stated but can be interpreted as a clinically detectable cavity [7].

Cariogram assesses the probability of *not* acquiring caries. The tool implies that not acquiring caries means having no dysbiosis with respect to dental caries or no detectable sign of incipient caries [8]. Accordingly, the outcome of interest in this tool is caries that is detectable by any means. We will come back to this definition of the outcome when talking about valuing the severity of outcomes. Here, we conclude that the outcome of interest is not explicitly stated. It is implicit, at most, as it is in CAMBRA.

### Uncertainty measures

The consumption of processed sugary foods does not with necessity cause caries, nor does having poor oral hygiene et cetera. However, population data suggest that these factors increase the chance to acquire caries, thereby making them risk factors [14–16]. Some risk factors are activities that can be controlled by the patient, others include conditions beyond the patients’ control. The likelihood that these risk factors lead to caries (or a more narrowly defined outcome) can be expressed quantitively (with probabilities) in Cariogram or qualitatively (as ‘Low’, ‘Medium,’ or ‘High’) in CAMBRA. Choosing one of these measures of uncertainty depends on how precise clinicians believe they can be in reporting the uncertainty. Importantly, the choice of measure must be based on what conveys the uncertainty most effectively to the clinician and patient.

Probabilities are not as straightforward as one might think. Even straightforward representations of probabilities, such as a 40% probability of caries, can be understood in different ways: It could mean that the patient will have caries in 40% of their teeth within two years’ time; that they will have 40% caries in all of their teeth; or that on a population level, 40% of all people with a certain set of risk factors will have caries [17]. Cariogram applies this last meaning, which the clinician must make the effort to communicate to the patient. Probabilities can also convey a false sense of security. Is the underlying evidence so robust that the Cariogram can confidently state a precise probability for not acquiring caries? Given the complex relationship between risk factors and caries incidence, perhaps a more imprecise presentation of the uncertainty is warranted, such as CAMBRA’s use of the qualitative measure of risk as ‘Low,’ ‘Medium,’ and ‘High’. The interpretation of these terms is however also problematic [18].

### Determining the severity of outcomes

When caries risk is assessed to inform decision making, the following questions are of relevance to the patient: How severe is the consequence? Are several cavities worse than one? How much worse is a cavity than an incipient lesion? Is slow progression as bad as fast progression? Scientific data to answer these questions are scarce. The risk assessment models that we have looked at do not include such answers either. Moreover, the answers are individually variable based on the individual perception of the clinician and patient.

The two models studied here, and this is probably common to many more, start from an assumption that it is sufficient to identify risk factors indicating that caries as a disease may occur in the future. Clinicians and patients can rely on prevalence and incidence numbers on a societal level—saving time, effort, and monetary costs for all involved parties. The speed of caries progression and the number of teeth affected are not the issue; rather, it is the plausibility that caries will occur that is of interest—the important thing being whether there is something there that needs management and intervention.

We believe that the above assumption is based on a dentist-centered perspective. From the patient’s point of view, what might be of most interest is the further consequences of having caries. For some, the smallest possibility of a tooth becoming fractured due to a carious lesion might be an unacceptable risk, while a high possibility of an incipient carious lesion progressing slowly is almost negligible. For others it could be the other way around.

If the risk assessment model only gives the probability of not acquiring caries, as in Cariogram, then the risk is the probability of detecting any sign of caries. Will an 80% probability of this warrant shortening the check-up intervals from two years to one? The answer depends on how the clinician and patient value the severity of the outcome. For example, based on the information from the Cariogram, the clinician may conclude that there is a medium risk at 40% chance of caries and propose a certain treatment accordingly; their decision depends on how they value caries (if caries were harmless, a 40% chance is not much of a risk; if it were deadly, a 40% chance would be a very high risk). The same goes for the patients. Patients base their valuation on how they understand caries, influenced by both how clinicians communicate it to them and how they see the world. Thus, there is not one, objective, value to set on the severity of consequences; the clinician may value one way, the patient another, and for matters of society or health economics, yet another valuation may seem appropriate. The prospect of a cavity in two years’ time may be unacceptable to the clinician, whereas patients may think it is perfectly acceptable since they trust the clinician to fix it once it develops. Therefore, clinicians need to explain the outcomes and the rationale behind their valuation clearly to the patient.

## Risk and risk communication

That risk involves both facts and values and has consequences for how the responsibility of dealing with risk is allocated. Experts assess the facts: identifying possible consequences of a risky activity, assessing the probability of their occurrence, and assessing the cost (in money, health, or discomfort) would the consequences occur. Decision makers then decide what to do given the information they get from the experts: they assess the costs of different consequences, the ability to afford said costs, and so on.

This division of labor is useful also when it comes to caries risk assessment. It can inform the function of a risk assessment tool and the way dentists handle caries risks in the dentist–patient interactions. Regarding caries risk, or risk of oral disease in general, the dentist (perhaps using a risk assessment tool) is the expert, and the patient is the decision maker. Psychologist and risk researcher Baruch Fischhoff has for many years pointed to the importance of risk communication [19,20]. He stresses that in order to make an informed decision, the decision makers (the patients) need to be informed about the facts that are relevant to them and be given these in a form that they understand. In the case of caries risks, this of course presupposes the nonpaternalistic view that patients are trusted to actually make their own decisions regarding their oral health.

The tool CAMBRA is an example that this is not always the case. The tool accounts for the risk and protective factors to allow the dentist to decide on a risk level; thereafter, the tool itself suggests a specific treatment plan. There appears to be no room for the patient’s input. In contrast, Cariogram shows more promise in that respect. It simply gives the probability of not acquiring caries, which is valuable, factual information for the dentist when communicating risk to the patient. However, as we have seen, there are problematic elements here, too. For example, if the relevant outcome is no detectable signs of caries, then the only thing that can be communicated based on the information from Cariogram is that there is an exact probability for some kind of caries in two (or more) years’ time. Is this sufficient for the patient to make an informed decision about what to do? Having degrees of possibilities for more individualized outcomes—such as developing a cavity, and losing a tooth, and having fast or slow progression—caters more to the needs of the patient.

## Final comments

No matter how relevant and well balanced the risk factors are, a caries risk assessment model may create problems if caries risk is not explicitly defined. We have mentioned above a couple of potential problems, for instance, clinicians having differing views on what the consequence of interest is. How to characterize uncertainty is another issue. Precise probabilities demand a very stable evidentiary basis. Without such a basis, the presentation of uncertainty should reflect the underlying evidentiary uncertainty using probability intervals or some qualitative expression of confidence (the Intergovernmental Panel on Climate Change provides a useful example) [21].

Furthermore, we argue that a risk assessment tool should not provide more than an assessment of the risk. That is, it should identify relevant, well-defined outcomes/consequences and provide a likelihood for their occurrence. It should not put values on the outcomes but rather provide scenarios of potential further outcomes (the latter may also preferably be a task for the treating dentist).

We have shown, with examples, that caries risk assessment models lack proper definitions of caries risk, and we have suggested aspects to consider when trying to define risk more clearly. Central to defining caries risk is having a clear grasp on what the outcome of interest is.

## CRediT author statement

Anna Senneby: Conceptualization, Visualization, Writing- Reviewing and Editing. Helena Fransson: Project administration, Visualization, Writing-Reviewing and Editing. Niklas Vareman: Conceptualization, Writing-Original draft preparation
